# The paradox of better population health after the pandemic: what is the cause?

**DOI:** 10.3389/fpubh.2025.1592366

**Published:** 2025-10-14

**Authors:** Marek Biernacki, Katarzyna Ostasiewicz

**Affiliations:** ^1^Department of Mathematics and Cybernetics, Wrocław University of Economics and Business, Wrocław, Poland; ^2^Department of Statistics, Wrocław University of Economics and Business, Wrocław, Poland

**Keywords:** excess death, COVID-19, HLY, public health, older people

## Abstract

**Objectives:**

This study aimed to verify the hypothesis that the improvement in the subjective assessment of population health in certain European countries after the COVID-19 pandemic was driven by the mortality of the majority of vulnerable citizens with the worst health status.

**Methods:**

We extended the trend of the share of the oldest age group and compared it with the observed fraction, thereby identifying the “missing population.”

**Results:**

We observed a substantial deficit in the population of the oldest age group, especially in countries where people tend not to age well.

**Conclusion:**

The temporary improvement in population health indicators, as measured by Healthy Life Years (HLY), during the pandemic in some countries was most likely an artifact resulting from the mortality of the majority of vulnerable individuals with poor health status. It is unlikely that this apparent improvement reflects healthier lifestyles or genuine gains in the efficiency or resilience of health systems during the pandemic. Therefore, the interpretation and use of HLY values from the COVID-19 period in Europe should be carefully reconsidered and further validated.

## 1 Introduction

The COVID-19 pandemic was the largest global health crisis since 1918, when the Spanish flu infected approximately 500 million people and caused over 50 million deaths (1). The Spanish flu primarily affected young and healthy individuals aged 15–45 years, with a mortality rate exceeding 10% of infections. By comparison, the COVID-19 pandemic infected over 600 million people and caused more than 6.5 million deaths, mostly among older individuals aged 65 years and above (2). In 1918, the infection rate was approximately 30%, while in 2020, it was 8%, nearly four times lower. Considering medical advances and increased public awareness over the last century, one might have expected a far milder impact from COVID-19.

An analysis of the Spanish flu pandemic in the USA (3) showed that areas hit harder experienced a sustained decline in economic activity. Conversely, cities that implemented non-pharmaceutical interventions (NPIs) such as social distancing and mask-wearing not only slowed transmission but also mitigated economic hardship, experiencing relative economic growth afterward.

OECD analyses of excess deaths in 2020–2021, compared to the five-year average, showed significant excess mortality during COVID-19 surges, especially in Poland, the Czech Republic, Slovakia, and Hungary—former Eastern Bloc countries—as well as Mexico, Colombia, and the United States (4, 5, 16). Another indicator of health system performance was the rate of unmet medical care needs, highest in Hungary, Portugal, Latvia, Poland, and the United States (17, 18). Overall, OECD reports concluded that health systems across countries struggled with efficiency and resilience during this period (6).

In addition to excess deaths and reduced access to care, COVID-19 created further health needs, including long COVID and cognitive impairments such as “COVID brain” (7–9). For instance, approximately 10% of patients reported symptoms beyond 3 days (10), and only 65% returned to their previous health within 14–21 days (11).

Two key indicators reflecting the population health impact of COVID-19 are self-perceived health status and Healthy Life Years (HLY). HLY captures both length and quality of life in relation to health, serving as a structural European health indicator. It is based on disability occurrence and mortality rates calculated using the Sullivan method through EU-SILC surveys. Notably, in Europe, men live shorter lives than women but experience relatively healthier years. HLY is part of the EU health indicators (ECHI), though it is not listed in the International Compilation of Health Indicators.

The pandemic’s effects were not limited to mortality alone. A literature review (12) identified numerous health complications emerging during and after the pandemic, known as long-term COVID-19 symptoms. These include the following:

- Fatigue as the most common musculoskeletal symptom
- Reduced exercise capacity
- Anxiety and depression as the most common mental health problems
- Prevalent physical and mental health issues among ICU survivors
- Disproportionate fatigue and mental health challenges among women

### 1.1 Research hypothesis

The increase in the HLY index during the COVID-19 pandemic was caused by the deaths of the oldest and the majority of chronically ill individuals.

### 1.2 Research questions

- How can the increase in the HLY index during the pandemic in certain European countries be interpreted?
- Where did subjective perceptions of health change during the pandemic compared to before, and to what extent?
- If these perceptions changed, what factors influenced this change?

## 2 Data

In the following analysis, we primarily used data from Eurostat (13), which covers European countries. Due to some data limitations, we focused on the EU-27 countries, as well as Norway, where data were available.

For excess mortality, we relied on data from OurWorldInData (2).

We considered 2019 as the last year before COVID-19 and 2022 as the year after the pandemic. Although the pandemic originated in 2019, it did not reach Europe until 2020, when the first cases were observed. While the pandemic was far from over in 2022—and, in fact, case numbers were increasing in 2023—the majority of countries declared the pandemic state and related lockdowns officially ended in 2022. Our analysis focused on changes in health indicators during the pandemic, specifically the year 2021.

## 3 Limited medical services during the pandemics and their consequences

During the pandemic, the European Foundation for the Improvement of Living and Working Conditions conducted an online survey: Living Conditions and Quality of Life. The results showed that healthcare institutions across Europe canceled or postponed services for patients not suffering from COVID-19 to cope with the surge of coronavirus infections requiring urgent care. According to the survey, 85% of respondents reported that a visit or treatment was unavailable due to the pandemic, 43% faced excessively long waiting times, and 37% did not receive medical care because they feared exposure to COVID-19 (17).

Not surprisingly, the proportion of the population reporting no unmet medical needs declined during the pandemic in 2020 and 2021. Compared to 2019, the average decrease across European countries was 1 percentage point in 2020 and 0.3 percentage points in 2021 (unweighted by country populations), or 0.3 and 0.4 percentage points, respectively, when weighted by the population size.

Another reason to focus on excess mortality rates rather than COVID-specific mortality is the challenge of accurately reporting COVID-19 deaths. Problems arose from differing methodologies between countries and even changes within countries over time. For example, *Health at a Glance 2021: OECD Indicators* (pp. 44–45) describes issues with the reliability of data on infections and deaths (OECD, 2021). Similarly, the article *Estimating excess mortality due to the COVID-19 pandemic* (19) confirmed that COVID-19 deaths were underestimated, and it remains unclear which deaths were directly attributable to SARS-CoV-2 infection versus those indirectly caused by the pandemic. Identical conclusions were drawn in the systematic literature review *The impact of the SARS-CoV pandemic on cause-specific mortality patterns* (14) and in *Tracking excess mortality across countries during the COVID-19 pandemic with the World Mortality Dataset* (4).

## 4 Changes in subjective health after COVID-19

**Figure 1:**
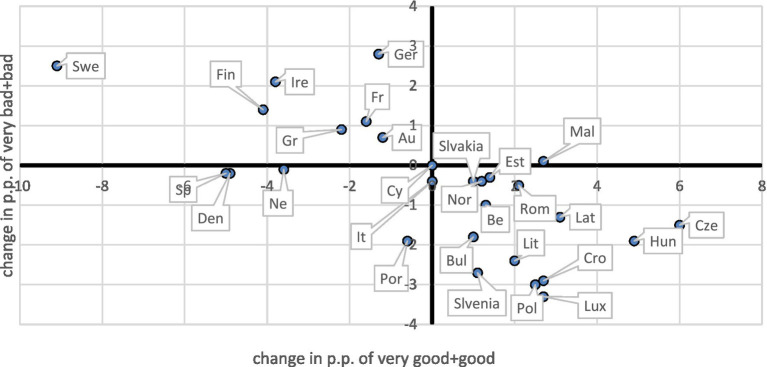
Changes in the fraction of the population in at least good and at most bad health in 2021 compared to 2019.

Using Eurostat data on subjective health from 2019 to 2022 (13), we calculated changes in the share of the population reporting their health as “very good” or “good” in 2021 and 2022 compared to 2019 (expressed in percentage points). We also analyzed changes in the share of individuals reporting their health as “bad” or “very bad.” These two measures are presented on a two-dimensional plot: the horizontal axis represents the change in the share of the population reporting very good or good health, while the vertical axis shows the change in the share reporting bad or very bad health. An increase along the horizontal axis is considered favorable, while a decrease along the vertical axis is also favorable. The results for 2021 are shown in Figure 1.

Depending on the quadrant in which a country appears on the plot, we defined four possible pandemic impacts on population health:

- *Improvement* (lower right): increase in very good or good health and decrease in bad or very bad health.
- *Deterioration* (upper left): decrease in very good or good health and increase in bad or very bad health.
- *Polarization* (upper right): increase in both very good or good and bad or very bad health.
- *Depolarization* (lower left): decrease in both very good or good and bad or very bad health.

A striking finding is that many countries fell into the *improvement* quadrant. Countries that remained in this quadrant in both 2021 and 2022 included Belgium, Bulgaria, Czechia, Estonia, Croatia, Latvia, Lithuania, Luxembourg, Hungary, Poland, Slovenia, Slovakia, and Norway. Conversely, Denmark and the Netherlands showed patterns of depolarization, while Germany, Ireland, and Sweden consistently appeared in the deterioration quadrant.

We also examined changes in Healthy Life Years (HLY) before and after the pandemic. In many countries, HLY increased, and this increase was positively correlated (*R*^2^ = 0.24) with perceived health improvements—an expected outcome, given the HLY construction. Notably, among the three countries with the largest decreases in HLY (Spain: −7.1, Sweden: −4.9, Denmark: −2.3), two (Sweden and Denmark) had excess mortality rates below 10% and pursued relatively less restrictive pandemic policies. Comparing these Nordic countries is particularly informative due to their similar cultural, economic, and healthcare systems.

In the following sections, we focused on self-perceived health and its determinants.

## 5 Relationships

It is somewhat surprising that population health improved in many countries after the pandemic. Moreover, the improvements were not necessarily observed in countries previously ranked highest in public health. To assess this, we constructed a provisional health index, *H*, defined as:


(1) $$H=2*f_{\mathrm{vg}}+f_{g}+0f_{f}-f_{b}-2f_{\mathrm{vb}}$$

where

$f_{\mathrm{vg}}$ —percent of the population who declare their state of health as “very good.”

$f_{g}$ —percent of the population who declare their state of health as “good.”

$f_{f}$ —percent of the population who declare their state of health as “fair.”

$f_{b}$ —percent of the population who declare their state of health as “bad.”

$f_{\mathrm{vb}}$ —percent of the population who declare their state of health as “very bad.”

The assigned weights in Equation (1) (2 for “very good,” etc.) may seem arbitrary, as self-perceived health is measured on an ordinal rather than an interval scale. However, for linear analysis, only the relative distances between categories matter. Treating the scale as symmetrical (with “good” and “bad” equally distant from “fair,” and the same for “very good” and “very bad”) seems reasonable, with the step between “good” and “fair” fixed at 1. The only arbitrary element is the choice of 2 for “very good.” We tested robustness by varying this weight between 1.5 and 2.5, finding qualitatively unchanged results, as discussed further on.

The differences H(2021)−H(2019) and H(2021)−H(2019) across European countries revealed that the top-ranking countries in both years included Croatia, Czechia, Hungary, Lithuania, Luxembourg, Poland, and Slovenia—the same countries previously identified in the improvement quadrant.

Next, we examined the relationship between health improvements and baseline (2019) health status, that is, regressions of H(2021)-H(2019) vs. H(2019) and H(2022)-H(2019) vs. H(2019). Both for H(2021)-H(2019) and H(2022)-H(2019), the slopes of regression were negative (−0.126 and −0.142, respectively) with coefficients of determination of 24 and 16% and *p*-values of 0.008 and 0.035, respectively. With a change of weight of the highest category between 1.5 and 2.5, slopes varied from −0.150 to −0.110 with determination from 29 to 20% for 2021 and from −0.170 to −0.122 with determination from 23 to 12% for 2022 (all significant with *p* < 0.05), being qualitatively the same.

On the other hand, the dependence of change of health on solely $f_{\mathrm{vb}}$ and $f_{b}$ was positive. For $f_{\mathrm{vb}}$ the linear relation was with $R^{2}=$ 0.23 with *p*-value 0.009 and $R^{2}=$ 0.23 with p-value 0.010 for 2021 and 2022, respecively. For $f_{b}$ the relation was with $R^{2}=$ 0.34 with p-value 0.001 and 0.28 with p-value 0.003 for 2021 and 2022, respectively. The strongest predictor was a combined “bad health index,” $H_{\mathrm{bad}}$ , defined as:


$$H_{\mathrm{bad}}=f_{b}+2f_{\mathrm{vb}},$$

with slope coefficients of 0.7353 and 0.9539 for 2021 and 2022, respectively, and $R^{2}=$ 0.32 (*p*-value 0.002) and 0.29 (*p*-value 0.003). Changing weights for the category “very good/bad” from 1.5 to 2.5 gave all positive slopes for 2021 $R^{2}$ from 0.18 to 0.32 (all significant with *p* < 0.05) and for 2022 $R^{2}$ from 0.13 to 0.30 (all significant with *p* < 0.05).

These findings suggest that the worse the state of health in a population before the pandemic, the more its average health indicators improved during COVID-19.

We estimated a linear model for relative health changes in 2021 (compared to 2019) as a function of baseline health, the share of the population with no unmet medical needs in 2020, and state health expenditures in 2021. The model results are given in Table 1. Although the overall model was significant (*p* = 0.01), only the baseline health variable was individually significant. Its coefficient was negative, indicating that populations with poorer health before the pandemic saw greater improvements, even after controlling for unmet needs and health spending during the pandemic.

**Table 1:** Results of the linear model for relative health changes in 2021 versus 2019, [H(2021)-H(2019)]/H(2019) expressed in standard deviations.

Independent variable	Estimation	*p*-value
Constant	−0.082	0.844
H(2019)	−0.002	0.007
No unmet needs 2020	0.004	0.121
Expenditures 2021	−3*10^−5^	0.382

All those relationships suggest that the improvement of population health may not be a real improvement of a given population but of the mortality of their weakest members, in the worst state of health.

## 6 Missing population

We hypothesized that excess mortality among the majority of vulnerable segments of the population—particularly older adults in poor health—may have altered the post-pandemic age structure. It is well established that self-perceived health generally declines with age. To examine this, we analyzed Eurostat (13) data on self-perceived health by age group (16–44, 45–64, 65–74, 75–84, 85+) in 2019 across European countries. On average, each additional year of age was associated with a 1.14 percentage point decrease in the share of people reporting very good or good health and a 0.42 percentage point increase in the share reporting bad or very bad health. These figures represent Europe-wide averages based on linear regressions fitted to health shares across age groups.

Table 2 provides the slope coefficients, interpreted as the expected change in percentage points per year of age, for each country. Here, $a_{\mathrm{vg}+g}\;$ denotes the slope of the regression line fitted to the share of the population declaring their health as very good or good, while $a_{\mathrm{vb}+b}\;$ refers to the slope of the line for the share declaring bad or very bad health. Higher absolute values of $a_{\mathrm{vg}+g}$ indicate a steeper age-related health decline, while higher $a_{\mathrm{vb}+b}$ values show a greater increase in poor self-perceived health with age. The determination coefficients (R^2^) are generally high (most exceeding 90%), with *p*-values mostly below 0.05, except in four countries for “bad+very bad,” where p-values remain below 0.1.

**Table 2:** Slope coefficients and determination coefficients of the regression lines for the share of persons with at least good and bad health, across increasing age groups.

Country	$a_{\mathrm{vg}+g}$	R2	$a_{\mathrm{bad}}$	R2
Belgium	−0.820	0.973	0.216	0.986
**Bulgaria**	**−1.545**	**0.979**	**0.596**	**0.846**
**Czechia**	**−1.419**	**0.994**	**0.512**	**0.879**
Denmark	−0.648	0.921	0.122	0.802
Germany	−1.014	0.980	0.343	0.737
**Estonia**	**−1.281**	**0.977**	**0.552**	**0.961**
Ireland	−0.513	0.990	0.107	0.782
Greece	−1.305	0.911	0.409	0.786
Spain	−1.166	0.962	0.377	0.892
France	−0.917	0.968	0.322	0.906
**Croatia**	**−1.447**	**0.979**	**0.701**	**0.970**
Italy	−1.255	0.946	0.457	0.828
Cyprus	−1.375	0.975	0.491	0.836
**Latvia**	**−1.229**	**0.961**	**0.748**	**0.922**
**Lithuania**	**−1.350**	**0.964**	**0.721**	**0.935**
Luxembourg	−0.877	0.987	0.283	0.930
**Hungary**	**−1.397**	**0.986**	**0.564**	**0.932**
Malta	−1.289	0.988	0.254	0.886
Netherlands	−0.694	0.934	0.110	0.817
Austria	−1.046	0.974	0.379	0.836
**Poland**	**−1.279**	**0.979**	**0.568**	**0.948**
**Portugal**	**−1.220**	**0.955**	**0.563**	**0.932**
**Romania**	**−1.533**	**0.969**	**0.536**	**0.868**
Slovenia	−1.160	0.994	0.489	0.829
**Slovakia**	**−1.474**	**0.994**	**0.621**	**0.940**
Finland	−0.920	0.961	0.244	0.812
Sweden	−0.671	0.844	0.118	0.913

Bold font indicates countries “bad for getting older”, i.e. with the absolute value of a_vg+g_ exceeding 1 and the value of a_vb+b_ exceeding 0.6.

Although age-related health deterioration is biologically expected, the rate of decline may vary across countries depending on the quality and resilience of their health systems to support aging populations. A smaller absolute value of $a_{\mathrm{vg}+g}$ suggests a less steep decline in health with age—indicating a country more supportive of healthy aging. Similarly, a lower value of $a_{\mathrm{vb}+b}$ suggests better healthy aging, as it indicates a smaller increase in the share of the population declaring very bad or bad health with age.

Arbitrarily, we defined countries as “bad for getting older” if the absolute value of *a_vg+g_* exceeded 1 and the value of *a_vb+b_* exceeded 0.6. According to this criterion, the following countries were identified: Bulgaria, Czechia, Estonia, Croatia, Cyprus, Latvia, Lithuania, Hungary, Poland, Portugal, Romania, and Slovakia. These countries also ranked among those with the greatest self-perceived health improvements after the pandemic.

Moreover, the relationship between $a_{\mathrm{vg}+g}$ and health improvements after COVID-19 was negative (which means, the better the country is for getting older—higher $a_{\mathrm{vg}+g}$ —the lower the health improvement after COVID-19), while between $a_{\mathrm{vb}+b}$ and heath change improvement, it is positive (again, it means that the worse the country is for getting older—the higher $a_{\mathrm{vb}+b}$ —the higher the improvement of the population’s health). The slope coefficients for regression of H(2021)-H(2019) on $a_{\mathrm{vg}+g}$ were negative and equal to −7.260 with $R^{2}=16\%$ , which was not very high, but the *p*-value was 0.032, so it was significant. In accordance, for $a_{\mathrm{vb}+b}$ , the slope was positive and equal to 10.162 with $R^{2}=26\%$ and a p-value of 0.006. Again, changing the absolute values of weights of categories “very good/bad” from 1.5 to 2.5, we obtained the results qualitatively similar, with $R^{2}$ ranging from 14 to 17% and significant at 0.05 for relation with $a_{\mathrm{vg}+g}$ , and with $R^{2}$ ranging from 25 to 26% and significant at 0.05 for relation with $a_{\mathrm{vb}+b}$ .

These findings support the interpretation that countries less favorable to healthy aging saw greater improvements in self-perceived health during the pandemic, possibly due to excess mortality among their oldest and the majority of vulnerable groups.

To further test this hypothesis, we introduced the concept of a *“missing silver population.”* Instead of simply checking whether the proportion of older adults declined (which was unlikely, given demographic trends), we tested whether their proportion increased less than expected based on pre-pandemic trends.

After evaluating various age groups, we selected the 85 + group due to its best minimum and average R^2^ values. We analyzed trend lines for 2013–2019 for each country and assessed their R^2^. For the 85 + group, the minimum R^2^ was 0.887 with an average of 0.977, compared to, for example, the 60 + group, which had values of 0.322 and 0.937, respectively. As the 85 + group showed the best predictability, we calculated the *missing population* in this group by comparing the expected share in 2022 (based on trends) with the observed share.

**Figure 2:**
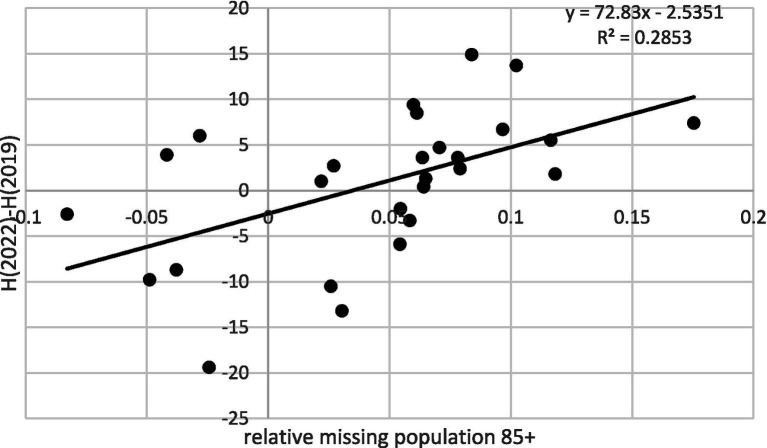
Health improvement after COVID-19 versus the relative missing population 85+.

Because the significance of one percentage point of missing population varies depending on whether the total 85 + group is 5% or 1% of the overall population, we calculated the *relative missing population* as a proportion of the expected size. The variability of this relative missing population explained nearly 38% of the variability in health changes (*p* = 0.001) (see Figure 2). If we excluded countries where observed values fell within the confidence intervals of the predictions, the determination coefficient even slightly increased to over 38% (*p* = 0.004). Robustness checks using weights from 1.5 to 2.5 for extreme categories of self-perceived health yielded determination coefficients ranging from 43% (*p* = 0.000) at a weight of 1.5 to 33% (*p* = 0.001) at a weight of 2.5, confirming the consistency of the findings.

This result strongly suggests that excess mortality during the pandemic, disproportionately affecting the majority of vulnerable members of society in the poorest state of health, contributed to the observed improvements in self-perceived population health during and after the pandemic. Given that health status generally deteriorates with age, this finding particularly concerns the oldest age groups.

## 7 Discussion of limitations

Without individual data, there is always a risk of so-called ecological fallacy; thus, we do not claim our results have the only possible interpretation, treating our interpretation rather as a suggestion.

## 8 Summary and conclusion

This article aimed to verify the hypothesis that the observed increase in subjective health assessments of European populations after the COVID-19 pandemic was driven, at least in part, by the mortality of the majority of vulnerable citizens with the poorest health status. Naturally, there are also other factors that could have contributed to improved self-perceived health.

These factors might include improved lifestyle, nutrition, and hygiene—determinants that, according to M. Lalonde, account for approximately 50% of health outcomes, or potential improvements in the quality of healthcare. However, nationwide quarantines and severe restrictions on mobility during the pandemic were unlikely to support healthier lifestyles. At the same time, healthcare resources were largely redirected to COVID-19 patients, leading to longer waiting times for specialist care, diagnostic tests, or hospital treatment. On the other hand, reduced social contact may have helped to limit the spread of other infectious diseases, such as influenza. From a psychological perspective, improvements in subjective health might have resulted from a sense of relief after surviving COVID-19 or avoiding its severe complications, thereby boosting mental resilience (15). Some ecologists have also argued that the reduction of industrial activity and pollution during the pandemic may have contributed to improved health, especially among individuals with circulatory or respiratory diseases.

Nonetheless, if even part of the observed improvement in health indicators was due to excess mortality among the weakest members of society, it raises an important question: is it justified to use the HLY index to assess changes in health status during the COVID-19 pandemic?

The data used in these analyses were taken from Eurostat and the Health Consumer Powerhouse, both relying on national statistical systems in high-income European countries with relatively similar cultural backgrounds. Therefore, the data can be considered high quality and consistent over the analyzed period. While COVID-19 mortality statistics themselves may be affected by reporting uncertainties, excess mortality during the pandemic is well-documented. Although self-reported health status can be subject to measurement error, especially during a crisis like a pandemic, using aggregate data for population-level assessments reduces the risk of ecological fallacy.

We acknowledge that these observations are far from conclusive and do not prove a causal relationship between excess mortality among the majority of vulnerable population and the improvement in overall population health indicators. We lacked access to individual-level data and can only infer, on average, that older adults who are “missing” from the data would likely have been in worse health. We also did not explore other possible demographic processes affecting the trend in the 85 + group. However, our methodological approach is comparable to the estimation of excess deaths, which is also based on relatively short trend projections.

In conclusion, since multiple relationships strongly suggest a link between excess mortality and the apparent improvement of health status, caution is warranted in interpreting indicators such as HLY as a measure of the *efficiency, resilience, or performance* of healthcare systems during the COVID-19 pandemic.

## Data Availability

Publicly available datasets were analyzed in this study. This data can be found at: https://ec.europa.eu/eurostat/data/database and https://ourworldindata.org/excess-mortality-covid.

## Appendix A

**Table A1:** Results for determination coefficients for trend 2013–2019 for 85 + population, predicted values for the time after COVID, and “missing population.”

Country	Observed share of 85 + population in 2021 [in percentage]	Predicted share of 85 + population (based on trend line) [in percentage]	Lower bound of 95% CI of prediction [in percentage]	Upper bound of 95% CI of prediction [in percentage]	Missing population 85 + [in percentage points]	Relative missing population 85 + [unitless]
Belgium	**2.873**	3.037	2.983	3.091	0.164	0.054
Bulgaria	2.100	2.129	2.084	2.175	0.029	0.014
Czechia	**1.923**	2.048	1.983	2.113	0.125	0.061
Denmark	**2.181**	2.113	2.093	2.133	−0.068	−0.032
Germany	**3.014**	2.870	2.737	3.002	−0.144	−0.050
Estonia	**2.700**	2.855	2.716	2.994	0.155	0.054
Ireland	**1.691**	1.638	1.595	1.682	−0.052	−0.032
Greece	**3.621**	3.768	3.672	3.863	0.146	0.039
Spain	**3.325**	3.487	3.413	3.561	0.162	0.046
France	**3.373**	3.503	3.436	3.569	0.129	0.037
Croatia	**2.369**	2.402	2.376	2.427	0.033	0.014
Italy	3.715	3.770	3.709	3.831	0.055	0.014
Cyprus	1.637	1.638	1.573	1.702	0.001	0.000
Latvia	**2.524**	2.673	2.543	2.803	0.149	0.056
Lithuania	**2.686**	2.817	2.759	2.875	0.131	0.047
Luxembourg	**1.945**	2.085	1.977	2.193	0.140	0.067
Hungary	**2.054**	2.136	2.094	2.177	0.082	0.038
Malta	1.962	1.954	1.914	1.994	−0.008	−0.004
Netherlands	**2.229**	2.278	2.234	2.322	0.049	0.021
Austria	**2.506**	2.618	2.545	2.692	0.112	0.043
Poland	**2.152**	2.289	2.199	2.378	0.136	0.060
Portugal	3.239	3.237	3.178	3.296	−0.002	−0.001
Romania	**2.142**	2.201	2.161	2.242	0.059	0.027
Slovenia	**2.588**	2.752	2.685	2.819	0.165	0.060
Slovakia	**1.558**	1.599	1.575	1.622	0.040	0.025
Finland	2.800	2.848	2.748	2.949	0.048	0.017
Sweden	2.531	2.526	2.485	2.567	−0.005	−0.002
Norway	2.179	2.178	2.149	2.207	−0.001	0.000
